# Case Report : Bilateral dentigerous cysts in association with developing third molars and premolars in a non-syndromic 13-year-old: a 2-year follow-up

**DOI:** 10.12688/f1000research.161532.1

**Published:** 2025-02-17

**Authors:** Osama Zakaria, Sami Alshehri

**Affiliations:** 1 Department of Biomedical Dental Sciences, College of Dentistry, Imam Abdulrahman Bin Faisal University, Dammam, Eastern Provence, 31441, Saudi Arabia

**Keywords:** dentigerous cyst, bilateral, mandible, pediatric, third molar, second premolar

## Abstract

**Background:**

Dentigerous cysts are benign, non-inflammatory odontogenic cysts that form over unerupted or partially erupted teeth. They are the second most common type of odontogenic cysts and can cause various complications if left untreated.

**Methods:**

We report a rare case of bilateral dentigerous cysts in the mandible of a 13-year-old boy. Radiographic examination revealed two cysts on each mandibular side, associated with developing third molars and unerupted permanent second premolars. Histopathological examination confirmed the diagnosis of dentigerous cysts.

**Results:**

The patient underwent surgical enucleation of all cysts and extraction of unerupted teeth under general anesthesia. The postoperative course was uneventful, with no recurrence observed over a 2-year follow-up period.

**Conclusions:**

Dentigerous cysts are rare in children and even rarer in bilateral presentation. Early diagnosis and treatment are essential to prevent potential complications such as infection, tooth displacement, jaw fracture, or malignant transformation. This case illustrates the importance of radiographic examination and histopathological confirmation in the management of dentigerous cysts.

## Introduction

Dentigerous cysts, benign and non-inflammatory odontogenic cysts, typically form over unerupted or partially erupted teeth and are the second most common type of odontogenic cysts after periapical cysts. ^1^ These developmental cysts, caused by fluid buildup between the reduced enamel epithelium and the tooth crown, predominantly occur in permanent dentition, commonly involving mandibular third molars, maxillary third molars, maxillary canines, and mandibular second premolars. ^2^ They are usually discovered in the second to fourth decades of life and are rare in childhood. ^3^

Typically painless, dentigerous cysts are often found during routine radiographic exams. However, they can grow large, causing swelling, tooth sensitivity, displacement, or a palpable mass. ^4^ Radiographically, they appear as well-defined, unilocular radiolucencies surrounding the crown of the affected tooth. ^5^ Histopathologically, they are lined by stratified squamous nonkeratinizing epithelium ^6^ Treatment depends on size and location, with surgical enucleation or marsupialization and tooth extraction being common. ^7^ Untreated, they can lead to infection, tooth loss, jaw fracture, or malignant transformation. ^8^

Bilateral dentigerous cysts in children are exceedingly rare. Our case involves a 12-year-old boy with bilateral dentigerous cysts in the mandible, presenting with painless swelling. This case is notable for two reasons: first, the presence of two cysts on each mandibular side, one associated with a developing third molar and the other with an unerupted permanent second premolar; second, the cysts exhibited histological features suggestive of an inflammatory origin, a rarity in dentigerous cysts. ^9^ This case’s uniqueness lies in its deviation from typical non-syndromic bilateral dentigerous cysts, contributing valuable insights to clinical practice and emphasizing the need for individualized treatment approaches. This report provides a rare insight into the long-term outcomes, specifically over a 2-year follow-up period, of treating bilateral dentigerous cysts in a pediatric patient.

## Case presentation

A 13-year-old boy presented at our clinic with a six-month history of painless swelling in the lower jaw. He had no significant past medical, surgical, or family history of dental anomalies or cystic lesions and was not on any medication.

**Figure 1.**
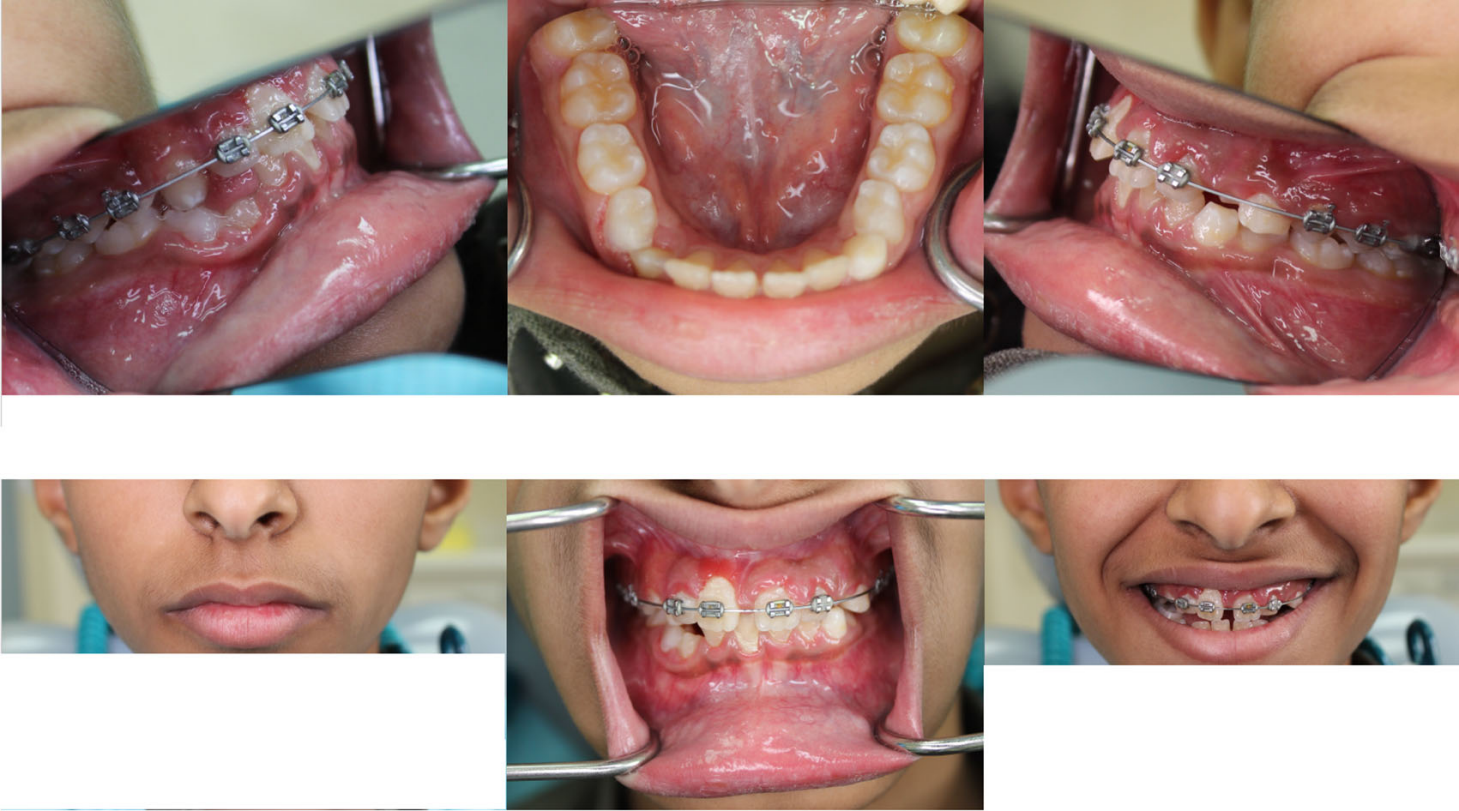
Bilateral mandibular swelling with normal skin and retained deciduous second molars.

Clinically, he exhibited bilateral symmetrical swelling in the mandibular body region, extending from the angle to the canine region, with normal overlying skin and mucosa. There was no tenderness, warmth, fluctuation, lymphadenopathy, or trismus ( Figure 1).

His vital signs were normal. Intraorally, he had mixed dentition with normal occlusion, retained deciduous mandibular second molars on both sides, and no caries, periodontal disease, or oral lesions. His oral hygiene was fair.

**Figure 2.**
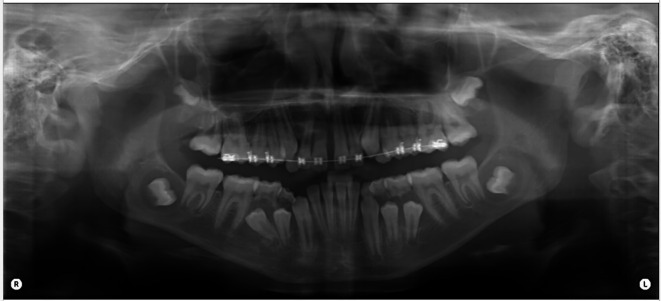
Preoperative panoramic radiograph (OPG).

Diagnostic assessment included panoramic (orthopantomograph OPG) and periapical radiographs and a CT scan of the mandible. The panoramic radiograph revealed four well-defined radiolucent lesions in the mandibular body region, associated with unerupted third molars and premolars bilaterally ( Figure 2).

**Figure 3.**
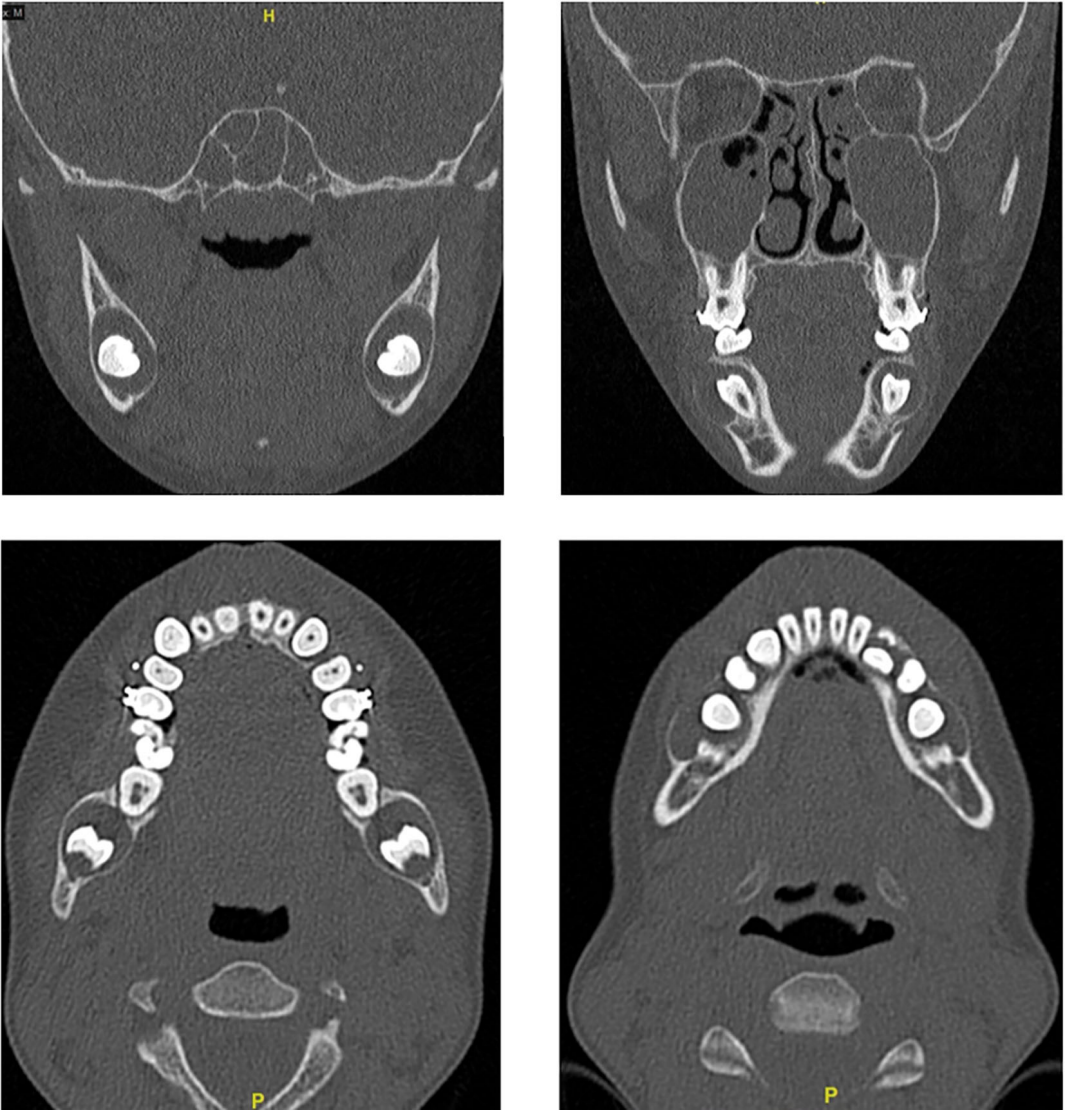
Preoperative CBCT showing unerupted teeth and bone expansion in coronal and axial views.

The CBCT scan showed unilocular, expansile lesions with thinning and bulging of the buccal and lingual cortices but no cortical breach or soft tissue extension ( Figure 3).

Laboratory blood tests and chest radiographs were normal. Genetic counseling ruled out syndromic associations like Gorlin-Goltz Syndrome or Maroteaux-Lamy Syndrome. The provisional diagnosis was bilateral dentigerous cysts, with differential diagnoses including odontogenic keratocyst, ameloblastoma, central giant cell granuloma, and unicystic ameloblastoma.

**Figure 4.**
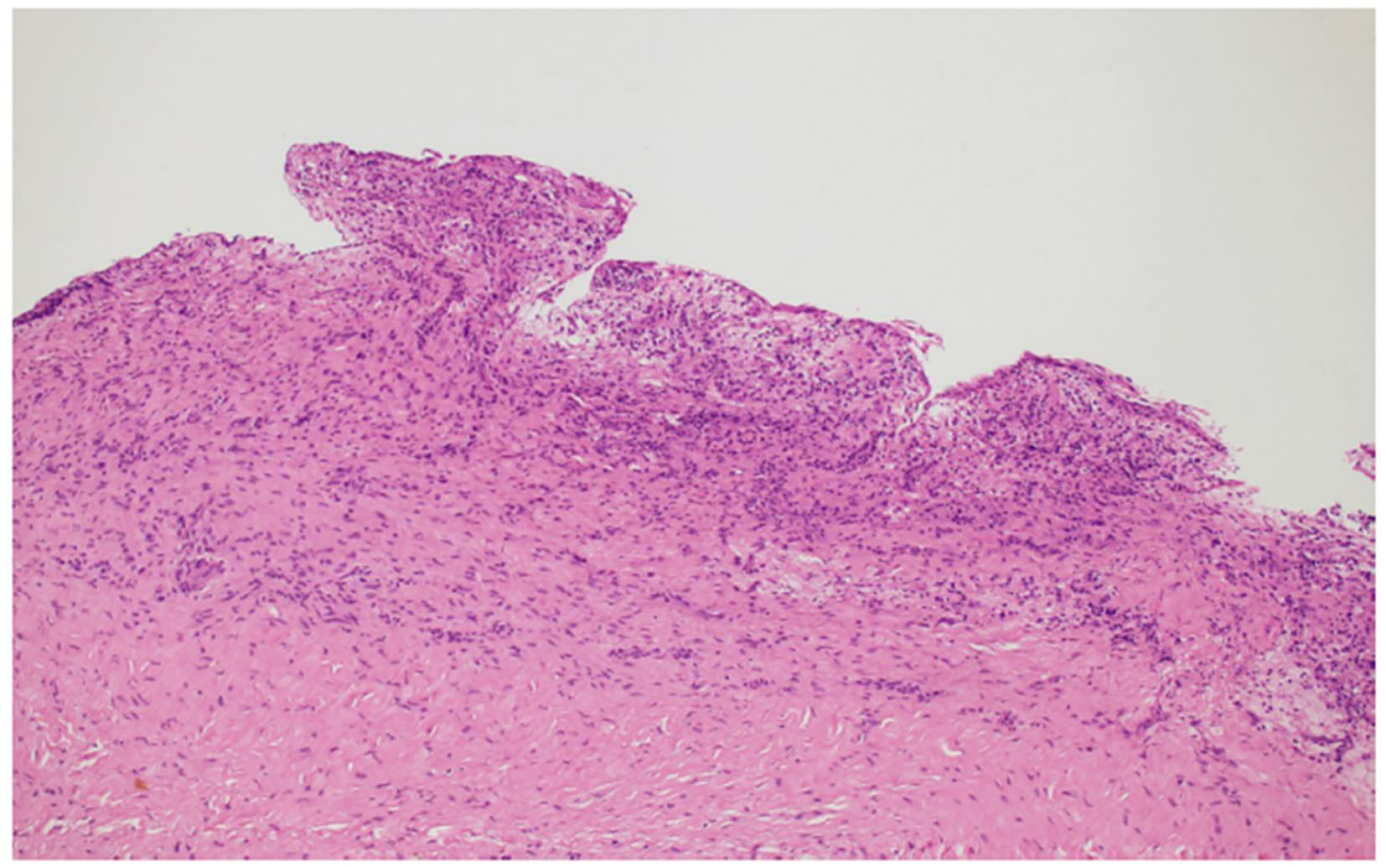
Photomicrograph showing thin cystic lining and fibrous stroma (H&E stain).

Histopathological examination showed cysts lined by stratified squamous nonkeratinizing epithelium with focal mucous cells, fibrous connective tissue with chronic inflammatory cells, and cholesterol clefts, but no dysplasia or malignancy ( Figure 4). This confirmed the diagnosis of dentigerous cysts.

**Figure 5.**
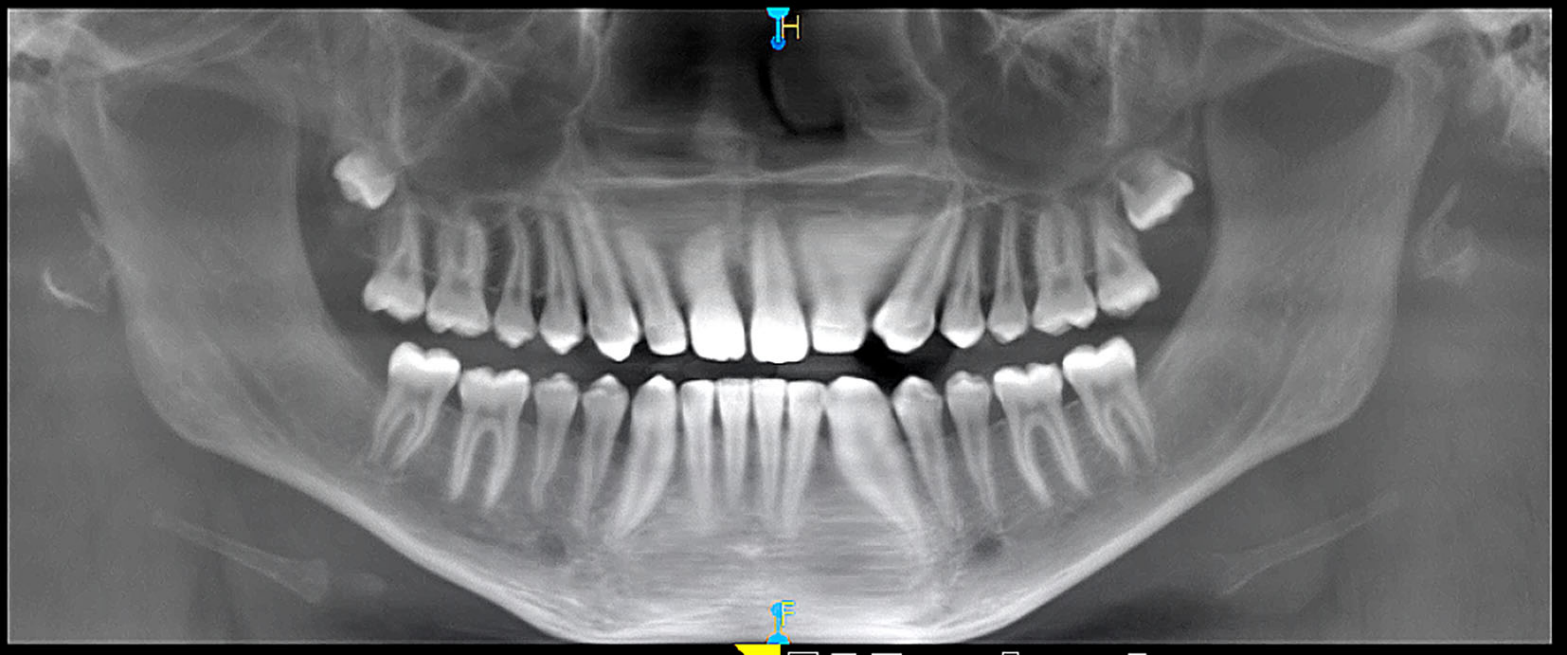
Panoramic radiograph (OPG) two years postoperatively.

**Figure 6.**
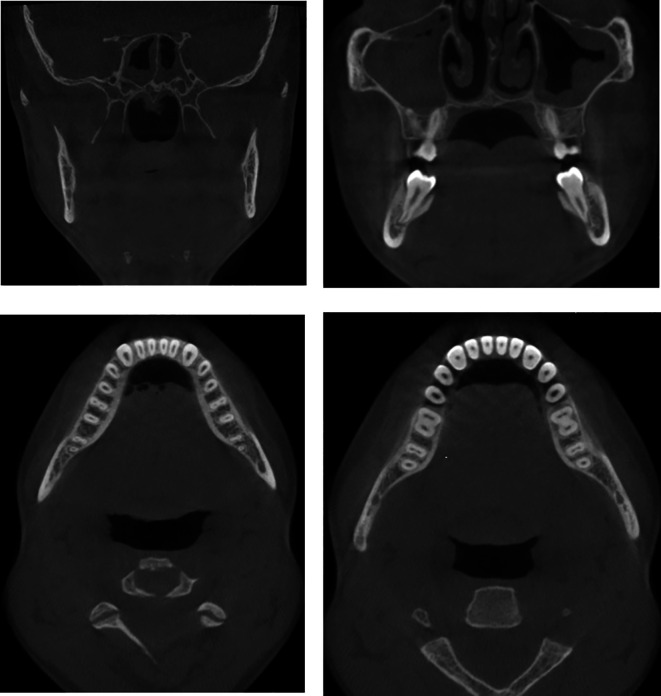
Postoperative CBCT showing erupted premolars, extracted molar site, and normal bone contour.

Therapeutically, the patient underwent surgical enucleation of both cysts and extraction of the developing third molars and retained primary teeth under general anesthesia. The surgical specimens were sent for histopathological examination. Postoperatively, he recovered well without complications, was prescribed antibiotics and analgesics, and advised on oral hygiene. Follow-up at one, three, six months, and continued monitoring over two years post-surgery showed passive eruption of lower premolars and complete root formation of lower premolars with bone regeneration at the lesion sites, with no recurrence or adverse effects ( Figures 5 & 6).

## Discussion

In the current case report, we meticulously detail the presentation of four dentigerous cystic lesions, which were observed bilaterally in the mandibular region. These lesions were notably associated with unerupted second premolars and third molars, with each of these teeth being implicated on both sides of the mandible. This bilateral occurrence of dentigerous cysts, particularly in association with both the second premolars and third molars, presents a unique and intriguing clinical scenario. The 2-year follow-up further validated the initial treatment plan’s success, as no recurrence or adverse effects were noted over the extended monitoring period.

In this case study, we detail the clinical encounter of a 13-year-old male patient, illustrating the broad age spectrum across which dentigerous cysts are documented. The literature is full with case reports that demonstrate the frequency of these cysts across a wide range of patient demographics. A one-year-old child serves as an example of a pediatric case, ^10^ while a 59-year-old woman serves as an example of an instance in the senior population. ^11^ This comprehensive presentation highlights the possibility that dentigerous cysts could develop at any stage of a person’s life. Notably, in pediatric cohorts, these cysts are frequently identified prematurely, commonly in association with unerupted permanent dentition. ^7^ Conversely, in adolescent and adult groups, the manifestation of dentigerous cysts typically correlates with the emergence of secondary dentition. ^1^

In the case study under discussion, the identified lesions were distinctly located bilaterally, affecting the premolars and third molars. It is noteworthy that bilateral dentigerous cysts occasionally manifest in unconventional sites, such as the incisula mandibulae, presenting concurrently and on both sides. ^12^ Anatomically, these cysts predominantly involve the mandibular region, particularly in proximity to impacted teetha characteristic consistently reported in numerous instances.

In this clinical narrative, the subject, a patient, manifested with a six-month history of non-painful swelling in the lower jaw region. Comparable literature illustrates that dentigerous cysts, often forming around unerupted mandibular first molars, are implicated in a spectrum of complications including pain, structural deformities, and displacement of teeth and nerve paresthesia. ^12^

It’s important to note that a significant portion of these cysts are unintentionally found during routine dental exams, as described by Aziz et al., ^3^ and Ozkan et al., ^13^ indicating that they usually don’t cause any symptoms. This pattern of incidental discovery is notably prevalent in pediatric and adolescent. In the current case study, the patient presented without any syndromic manifestations. Similar to our case, certain occurrences, such as the one documented by Esmaelizadeh et al., ^15^ are observed in non-syndromic patients.

In this case, the patient presented without any syndromic manifestations, consistent with other non-syndromic cases in the literature. For example, Esmaelizadeh et al. ^15^reported a non-syndromic case, while other reports, like Vinereanu et al., describe an autistic patient with bilateral odontogenic cysts around lower second molars. Additionally, Catarina et al. ^14^ documented homozygous twins with dentigerous cysts linked to unerupted lower second molars, one of whom also had a cyst associated with an unerupted maxillary canine. Some cases are associated with syndromes Rai et al. ^16^ and Roberts et al. ^17^ Batra et al. ^18^ even identified chromosomal polymorphism, suggesting a genetic component in certain instances.

In this case radiographic investigation, delineated four distinct, unilocular, expansile radiolucent entities within the mandibular body, characterized by buccal and lingual cortical thinning and protrusion, albeit without any evident cortical violation or extension into the surrounding soft tissues. The imperative role of radiographic assessment in differentiating dentigerous cysts from analogous pathologies, such as periapical cysts, is underscored. ^19^ The radiographic attributes of dentigerous cysts, as explicated in these analyses, include their oval morphology, ^19^ unilocular configuration, ^20^ radiolucency with corticated or radiopaque demarcation, and their influence on the mandibular corpus and the inferior mandibular canal. ^19^

In the described case, the patient underwent surgical enucleation of both cysts along with the extraction of developing third molars and retained primary teeth under general anesthesia. In contrast, other literature emphasizes a more conservative approach; marsupialization is often advocated to conserve permanent teeth and surrounding tissues. ^11, 12^ Some practitioners opt for enucleation of smaller lesions and marsupialization of larger ones, aiming to preserve permanent teeth and secure optimal final tooth positioning. ^9^ These practices highlight the importance of conservative treatment strategies in bilateral dentigerous cysts to facilitate proper bone healing and tooth eruption. Notably, the disease’s progression and clinical management approaches can vary, exemplified by Shah et al., ^4^ who reported a rare case of spontaneous cyst regression. Clinical management spans a spectrum from conservative measures, as seen in reports by Chew ^5^and Shah, ^4^ to surgical interventions such as enucleation, extraction, ^3, 13^ or marsupialization ^21^ Treatment modality may also be influenced by patient age, with conservative management preferred for younger children to preserve the developing dental and jaw structures, ^9, 21, 22^ while surgical intervention is more prevalent in adolescents facing larger cysts or more severe complications. ^8^

### Limitations

The limitations In this case report that shares with almost all dentigerous cysts reports in that it describes only a single, limiting the ability to generalize findings to a broader population.

## Conclusions

This case highlights the successful treatment of bilateral dentigerous cysts in a 13-year-old, with no recurrence observed over a 2-year follow-up. Early diagnosis and surgical management proved effective in preventing complications.

## Consent to publish

Written informed consent for publication of this case report and accompanying images was obtained from the patient’s legal guardian.

## Data Availability

No data are associated with this article.
