# Supplementary material for: Case Report : Bilateral dentigerous cysts in association with developing third molars and premolars in a non-syndromic 13-year-old: a 2-year follow-up
Source: F1000Res. 2025 Feb 17;14:215. [Version 1] doi: 10.12688/f1000research.161532.1 (PMC12120407; doi:10.12688/f1000research.161532.1)
Supplement: Peer review history

# Reviewer report: Reviewer response for version 1

**DOI:** 10.5256/f1000research.177571.r379989

**Published:** 2025-05-28

**Authors:** Georges Aoun

**Affiliations:** 1 Lebanese University, Beirut, Lebanon

This case report describes rare non-syndromic bilateral dentigerous cysts in a 13-year-old patient. It highlights well the clinical, radiological, and histopathological presentations as well as the management and outcomes of such a rare case.

 I would, however, like to suggest minor comments:

 1. In the introduction, the authors note that the size and position of the cyst determine the course of therapy; other factors must be taken into consideration, like the patient's age and the possibility that the affected tooth can be saved.

 2.The authors note in the introduction that bilateral dentigerous cysts in children are rare. They must add **non-syndromic** since bilateral and multiple cysts are frequently observed in connection with several syndromes.

 3. No aspiration was done?

 4. Add arrows on the CBCT images to illustrate the findings.

 5. Provide further details on the histopathological section and include arrows to illustrate the results.

Are enough details provided of any physical examination and diagnostic tests, treatment given and outcomes?

Yes

Is the case presented with sufficient detail to be useful for other practitioners?

Yes

Is sufficient discussion included of the importance of the findings and their relevance to future understanding of disease processes, diagnosis or treatment?

Yes

Is the background of the case’s history and progression described in sufficient detail?

Yes

Reviewer Expertise:

Oral Medicine

I confirm that I have read this submission and believe that I have an appropriate level of expertise to confirm that it is of an acceptable scientific standard.

# Reviewer report: Reviewer response for version 1

**DOI:** 10.5256/f1000research.177571.r368658

**Published:** 2025-02-28

**Authors:** Ana Cláudia Garcia Rosa

**Affiliations:** 1 Federal University of Tocantins, Palmas, Brazil

This article presents a well-documented case report of bilateral dentigerous cysts associated with developing third molars and premolars in a non-syndromic 13-year-old. The images are good quality, and the writing is clear and informative. However, I have minor suggestions for improvement.

 Introduction:

1. The last paragraph of the introduction contains some repetition and redundancies, which give the impression of AI-generated writing. I recommend that the authors revise this section for clarity, ensuring a more concise and natural flow.

Case presentation:

1. In the phrase, “Diagnostic assessment included panoramic (orthopantomograph OPG) and periapical radiographs and a CT scan of the mandible,” consider inserting a comma after “orthopantomography” or rephrasing the sentence for better clarity. A possible revision is: “Diagnostic assessment included orthopantomography (OPG), periapical radiographs, and a CT scan of the mandible.”

Discussion:

1. There is a punctuation issue in the following sentence: “Some cases are associated with syndromes Rai et al.16 and Roberts et al.17 Batra et al.18 even identified chromosomal polymorphism, suggesting a genetic component in certain instances.” Proper punctuation should be added.

Limitations:

1. The sentence discussing the limitations of the case report is unclear and contains grammatical issues. I recommend revising it for better clarity. A possible revision is: “The main limitation of this case report, as with most reports on dentigerous cysts, is that it describes only a single case, which limits the ability to generalize findings to a broader population.”

Final suggestion:

 All information should be carefully verified if the authors used AI assistance to construct the manuscript.

Are enough details provided of any physical examination and diagnostic tests, treatment given and outcomes?

Yes

Is the case presented with sufficient detail to be useful for other practitioners?

Yes

Is sufficient discussion included of the importance of the findings and their relevance to future understanding of disease processes, diagnosis or treatment?

Yes

Is the background of the case’s history and progression described in sufficient detail?

Yes

Reviewer Expertise:

Oral Pathology

I confirm that I have read this submission and believe that I have an appropriate level of expertise to confirm that it is of an acceptable scientific standard.
